# Concentrations and sources of heavy metals in shallow sediments in Lake Bafa, Turkey

**DOI:** 10.1038/s41598-020-68833-2

**Published:** 2020-07-16

**Authors:** Fulya Algül, Mehmet Beyhan

**Affiliations:** Department of Environmental Engineering, Faculty of Engineering, Süleyman Demirel University, Isparta, Turkey

**Keywords:** Environmental impact, Limnology

## Abstract

The concentrations and sources of heavy metals in shallow sediments in Lake Bafa were investigated. The concentrations of nine heavy metals and the total organic carbon content in sediment samples were determined for between Summer 2015 and Spring 2016. The mean contents of heavy metals were in decreasing order Fe > Mn > Ni > Cr > Zn > Cu > Co > Pb > Cd. Sediment quality guidelines indicate that Cr, Cu, and Ni pose a considerable threat to the aquatic ecosystem in Lake Bafa. Site L3 was found to be contaminated with Cd, Cr, and Ni, and the pollution load indices suggest that these metals had anthropogenic sources. The sediment samples were notably enriched with Cd and Ni. There is no consistent trend for seasonal effect in terms of the sample locations. However, at all sampling points, an increase in heavy metal concentrations was observed in the autumn. The results of a multivariate analysis indicate that the sources of Co, Cu, Fe, Mn, Pb, and Zn were all natural, the sources of Cd were anthropogenic, and the sources of Ni and Cr were both anthropogenic and natural. These results highlight that Cd, Cr, and particularly Ni represent the most serious threat in terms of heavy metal pollution in the ecosystem of the lake.

## Introduction

Heavy metals mainly enter aquatic environments as a result of a variety of human activities (e.g., agriculture, combustion, corroded underground pipes, industrial plants, sewage, smelting, and vehicles)^1–3^. Exposure to heavy metals has been linked to various negative health effects, including cancer, behavioural problems, impaired intelligence, developmental problems, kidney damage, and miscarriage or stillbirth^4^. Heavy metals are not readily degraded in the environment, and those that enter a water body can remain there for some time. They are usually found in low concentrations in aquatic systems^5^, and high concentrations of heavy metals in sediments can indicate anthropogenic rather than natural sources^6,7^.

Heavy metals are poorly soluble in water, so predominantly sorb to suspended particles that then settle as sediment^8^. Heavy metals can thus enter the food chain in the aquatic environment, and become available for accumulation in biota^9^. Fish, which are at the top of the food chain and are an important food source for humans, can accumulate heavy metals in their tissues^10^, and this characteristic makes them an effective indicator of pollution^11^. Accordingly, assessment of pollution from heavy metals in sediments in the environment is very important in terms of its effects on aquatic organisms and human health^12^. Lake sediments are a sink for heavy metals^13,14^, and heavy metal concentrations are generally higher in sediment than in water^15^. Indeed, concentrations of heavy metals in water are sometimes lower than detection limits, meaning that sediment should be analysed to assess levels of contamination by heavy metals in the aquatic system^16–18^.

Lake sediments mostly act as a sink for heavy metals; on the other hand, they can also act as a source to the overlying water^19,20^. Contaminated sediments can act as non-point sources of heavy metals to the water column^21^ when the chemistry of the aquatic system changes, for example if certain biochemical processes occur, if organic complexing agents enter the system, if the pH changes, if the redox conditions change, or if the salinity increases^22,23^. The release of heavy metals from sediments to the overlying water causes secondary pollution and can cause significant damage to the ecological status of the aquatic system^20,24^.

The degree of heavy metal pollution of sediment and the risks posed by the heavy metals therein can be assessed using methods of geochemical normalization such as sediment quality guidelines (SQGs), geoaccumulation indices (I_geos_), enrichment factors (EFs), contamination factors (CFs), and pollution load indices (PLIs)^25–27^. Heavy metal pollution in sediment is often assessed using statistical methods such as Pearson correlation analysis, principal component analysis (PCA), factor analysis, and hierarchical cluster analysis (HCA)^28–30^.

As a nature reserve, Lake Bafa is one of the most important lakes in Turkey, and has unique ecological characteristics. In the past few decades, however, pollution has caused a deterioration in the quality of the water and sediment in Lake Bafa. The main sources of pollutants are agriculture, the Büyük Menderes River, runoff from settlements without sewage treatment systems, untreated wastewater from aquaculture facilities and olive oil mills, and wastewater from tourist facilities^31^. The study area is influenced by intensive fishing. Therefore, heavy metals constitute a significant threat to human health and all living organisms. Given that Turkey lies under the influence of the European Union (EU), efforts have been made to address the aims of the Water Framework Directive 2000/60/EC (WFD)^32^. These efforts are increasing the significance of monitoring and assessment, especially in sediments and biota in surface water resources in Turkey. Building on existing literature, a limited number of studies have been undertaken on heavy metal pollution in the sediments of Lake Bafa^33–36^. Previous studies in the area have generally been focused on spatial changes in heavy metal pollution, and the distribution of heavy metals within the soil structure. In the present study, the degree of pollution and the sources of heavy metals in the sediment in what is a relatively dynamic system have been examined both spatially and seasonally in order to obtain a more up-to-date understanding of the environment. In addition, heavy metal contamination in the inflow (C1) and outflow (C2) channels of Lake Bafa has been assessed for the first time, thereby allowing a better understanding of the degree of heavy metal pollution in sediments both in the lake itself and in the channels leading to and from it. The levels of heavy metal pollution, the spatial distribution, and the risks posed by heavy metals in sediments in the lake have received little attention to date. The data on the risks posed by heavy metals in sediments in Lake Bafa discussed herein are expected to be of use to researchers and legislators in environmental management. The specific aims of the study were therefore (i) to determine the concentrations and spatial distributions of Cd, Co, Cr, Cu, Fe, Mn, Ni, Pb, and Zn in shallow surface sediments in Lake Bafa; (ii) to use geochemical normalization with CF, EF, I_geo_, and PLI to assess the heavy metal pollution of sediments in Lake Bafa and to identify the risks they pose in the sediment; (iii) to investigate heavy metal pollution in sediments in Lake Bafa using multivariate analytical methods.

## Results

### Sediment quality guidelines (SQGs)

In developing countries in general and in Turkey in particular, ecotoxicological assessments and studies to determine background values are scarce, especially for sediments. Therefore, for the purposes of this study, globally accepted SQGs were used instead of ecotoxicological results^37–40^, and Average Shale Values (ASVs) were used instead of background values^41–45^. SQGs include criteria designed to assess sediment quality and the ecological risks associated with heavy metals^46^. The most widely used forms of empirical SQGs are based on sediment chemistry and biological effects, and on mechanistic techniques related to equilibrium partitioning^47^. To assess sediment pollution, researchers have developed several SQGs using large data sets^48^. One of the most important methods developed in this area belongs to Long and MacDonald^49^ and MacDonald et al.^50^, who suggest “consensus-based” SQGs for assessing the environmental effects of metals. Consensus-based SQGs are determined as the geometric mean of different SQGs^51^, including those obtained using US-EPA and other similar guidelines^52^. Consensus-based SQGs contain two effect values, namely the threshold effect concentration (TEC) and the probable effect concentration (PEC). If the heavy metal concentrations measured in the sediment are below TEC, heavy metals are not expected to have any adverse effects on organisms. However, if the heavy metal concentrations in the sediment are above PEC, toxic effects are likely to occur^50^. As an alternative, the sediment environmental quality standards (EQSs) for heavy metals are not specifically defined in the WFD. EQS values for sediments have been determined only in a few countries, but are not yet available for Turkey. For this reason, we used consensus-based SQGs to assess the contamination of heavy metals of shallow sediments in Lake Bafa. We also compared the heavy metal concentrations in shallow sediments of the lake with background values. ASVs reported by Turekian and Wedepohl 1961^53^ and Average Crustal Values (ACVs) given by Taylor 1964^54^ are commonly used as background values in the evaluation of sediment pollution. Natural background heavy metal concentrations are not available for sediment in Lake Bafa, therefore ASVs were used as background values. The heavy metal concentrations in the shallow sediment samples and guideline concentrations are summarized in Table 1. The Cd, Cr, Co, Cu, Pb, Mn, Ni, and Zn concentrations were 0.400–3.92, 18.90–120.00, 6.15–20.50, 8.10–35.20, 5.60–21.30, 247.00–584.00, 46.30–251.00, and 20.70–44.30 mg/kg, respectively, and the Fe concentrations were 12.60–36.20 g/kg. The highest seasonal mean Co, Cu, Mn, Ni, and Zn concentrations (18.35, 35.50, 557.25, 195.00, and 42.05 mg/kg, respectively) were found in sediment from location L2, and the highest seasonal mean Cd, Cr, and Fe concentrations (1.31 mg/kg, 88.95 mg/kg, and 32.85 g/kg, respectively) were found in sediment from L3. The highest seasonal mean Pb concentration (16.15 mg/kg) was found in sediment from C1.

**Table 1:** Descriptive statistics for the heavy metal concentrations in Lake Bafa shallow sediment.

Heavy metals	Sampling points	Seasons					ASV	TEC	PEC
		Summer	Autumn	Winter	Spring	Seasonal mean ± SD			
Cd (mg/kg)	L1	0.4	0.42	0.4	0.4	0.41 ± 0.01	0.3	0.99	4.99
	L2	0.48	0.52	0.4	0.4	0.45 ± 0.06			
	L3	0.52	3.92	0.4	0.4	1.31 ± 1.74			
	L4	0.4	0.44	0.47	0.4	0.43 ± 0.03			
	C1	2.63	0.4	0.4	0.4	0.96 ± 1.12			
	C2	0.4	0.4	0.4	0.4	0.40 ± 0.00			
	Mean ± SD	0.81 ± 0.90	1.02 ± 1.42	0.41 ± 0.03	0.40 ± 0.00				
Cr (mg/kg)	L1	66.4	120	55.5	56.8	74.68 ± 30.61	90	43.4	111
	L2	93	86.2	81.2	92.7	88.28 ± 5.66			
	L3	88.9	87.9	92.3	86.7	88.95 ± 2.41			
	L4	72	97.5	89	39.4	74.48 ± 25.67			
	C1	58.4	58.4	65.3	60.7	60.70 ± 3.25			
	C2	76.7	35.8	33.5	18.9	41.23 ± 24.81			
	Mean ± SD	75.90 ± 13.22	80.97 ± 29.74	69.47 ± 22.57	59.20 ± 27.94				
Co (mg/kg)	L1	13.3	20.5	11.1	10.5	13.85 ± 4.59	19	NG	NG
	L2	19	18.5	17.4	18.5	18.35 ± 0.68			
	L3	18.2	18.1	18.5	18	18.20 ± 0.22			
	L4	16	19.8	18.8	9.52	16.03 ± 4.63			
	C1	13.4	14.5	15.6	16.2	14.93 ± 1.24			
	C2	15.7	6.15	8.13	6.54	9.13 ± 4.46			
	Mean ± SD	15.93 ± 2.36	16.26 ± 5.37	14.92 ± 4.36	13.21 ± 5.01				
Cu (mg/kg)	L1	10.3	20.5	9.5	8.1	12.10 ± 5.67	45	31.6	149
	L2	34.2	35.6	35.2	37	35.50 ± 1.16			
	L3	32.2	31.8	31.5	32.6	32.03 ± 0.48			
	L4	29.2	35.2	33.8	11.9	27.53 ± 10.73			
	C1	11.6	16.7	16.7	18.3	15.83 ± 2.92			
	C2	19.9	11.9	10.3	9.4	12.88 ± 4.80			
	Mean ± SD	22.90 ± 10.48	25.28 ± 10.23	22.83 ± 12.01	19.55 ± 12.40				
Fe (g/kg)	L1	19.9	34.6	17	15.9	21.85 ± 8.67	47.2	NG	NG
	L2	33.4	32.8	31	33.2	32.60 ± 1.10			
	L3	32.7	33	33.1	32.6	32.85 ± 0.24			
	L4	26.8	36.2	32.6	15.6	27.80 ± 9.01			
	C1	21.1	23.8	24.5	25.5	23.73 ± 1.88			
	C2	25.3	12.6	17.5	14.8	17.55 ± 5.54			
	Mean ± SD	26.53 ± 5.66	28.83 ± 9.05	25.95 ± 7.41	22.93 ± 8.66				
Pb (mg/kg)	L1	8	9.4	5.7	5.6	7.18 ± 1.85	20	35.8	128
	L2	14.6	15.8	15.1	14.4	14.98 ± 0.62			
	L3	14.2	14	13.9	14.5	14.15 ± 0.26			
	L4	13.1	15.6	15.3	8	13.00 ± 3.51			
	C1	10.4	21.3	20.6	12.3	16.15 ± 5.60			
	C2	9.5	6.4	8.6	5.9	7.60 ± 1.73			
	Mean ± SD	11.63 ± 2.71	13.75 ± 5.25	13.20 ± 5.30	10.12 ± 4.12				
Mn (mg/kg)	L1	360	455	247	255	329.25 ± 98.38	850	NG	NG
	L2	570	540	535	584	557.25 ± 23.60			
	L3	534	513	520	520	521.75 ± 8.81			
	L4	447	576	512	339	468.50 ± 101.13			
	C1	330	435	420	420	401.25 ± 48.02			
	C2	417	293	306	413	357.25 ± 66.91			
	Mean ± SD	443.00 ± 94.62	468.67 ± 100.75	423.33 ± 122.10	421.83 ± 118.92				
Ni (mg/kg)	L1	125	251	102	99.7	144.43 ± 71.96	68	22.7	48.6
	L2	203	192	184	201	195.00 ± 8.76			
	L3	193	193	199	192	194.25 ± 3.20			
	L4	156	210	198	81.5	161.38 ± 58.07			
	C1	149	138	159	158	151.00 ± 9.76			
	C2	158	71.8	72.4	46.3	87.13 ± 48.79			
	Mean ± SD	164.00 ± 29.01	175.97 ± 62.65	152.40 ± 53.35	129.75 ± 63.16				
Zn (mg/kg)	L1	25.2	39.6	21.6	20.8	26.80 ± 8.75	95	121	459
	L2	40.2	42.7	43	42.3	42.05 ± 1.27			
	L3	41.1	40	41.1	40.7	40.73 ± 0.52			
	L4	35.4	44.3	42.5	20.7	35.73 ± 10.73			
	C1	29	31.3	31.8	34.9	31.75 ± 2.43			
	C2	36.1	21.2	28.7	15.2	25.30 ± 9.07			
	Mean ± SD	34.50 ± 6.26	36.52 ± 8.74	34.78 ± 8.79	29.10 ± 11.62				

SD: standard deviation; ASV: average shale value^53^; TEC: threshold effect concentration; PEC: probable effect concentration^50^; NG: no guideline.

There is no consistent trend in seasonal effect for any of the sample locations. An increase in heavy metal concentrations was observed at all sampling points in the autumn, although the maximum spatial mean Cd, Cr, Co, Cu, Fe, Pb, Mn, Ni, and Zn concentrations (1.02 mg/kg, 80.97 mg/kg, 16.26 mg/kg, 25.28 mg/kg, 28.83 g/kg, 13.75 mg/kg, 468.67 mg/kg, 175.97 mg/kg, and 36.52 mg/kg, respectively) were observed at this time.

Heavy metal concentrations in the shallow sediment samples are shown in the boxplots in Supplementary Fig. S1 online, with the guideline concentrations also indicated. The seasonal mean Fe, Pb, Mn, and Zn concentrations for all sampling points were lower than the respective ASVs. The seasonal mean Cd, Co, and Ni concentrations were higher than the ASVs. The seasonal mean Cr, Cu, and Ni concentrations for most of the sampling points were higher than the TECs. The seasonal mean Cr concentrations for all the sampling points except C2 were higher than the TEC, as were the seasonal mean Cu concentrations for L2 and L3. The seasonal mean Ni concentrations for all the sampling points were all higher than the PEC. Cd concentrations for L3 and C1 were higher than the TEC in autumn and in summer, respectively. Cr concentrations were higher than the PEC in autumn. The SQGs indicate that Cd, Cr, Cu, and particularly Ni pose a serious threat to the ecosystem in Lake Bafa.

### Sediment pollution indices

Various pollution indices for assessing heavy metal pollution in sediment have been developed, and these indices have been successfully used in numerous studies^55–58^. We used I_geo_, EF, CF, and PLI to assess heavy metal pollution in Lake Bafa. Natural background heavy metal concentrations in Lake Bafa sediment are not available, so ASVs from previous publications were used to calculate the pollution indices.

**Figure 1:**
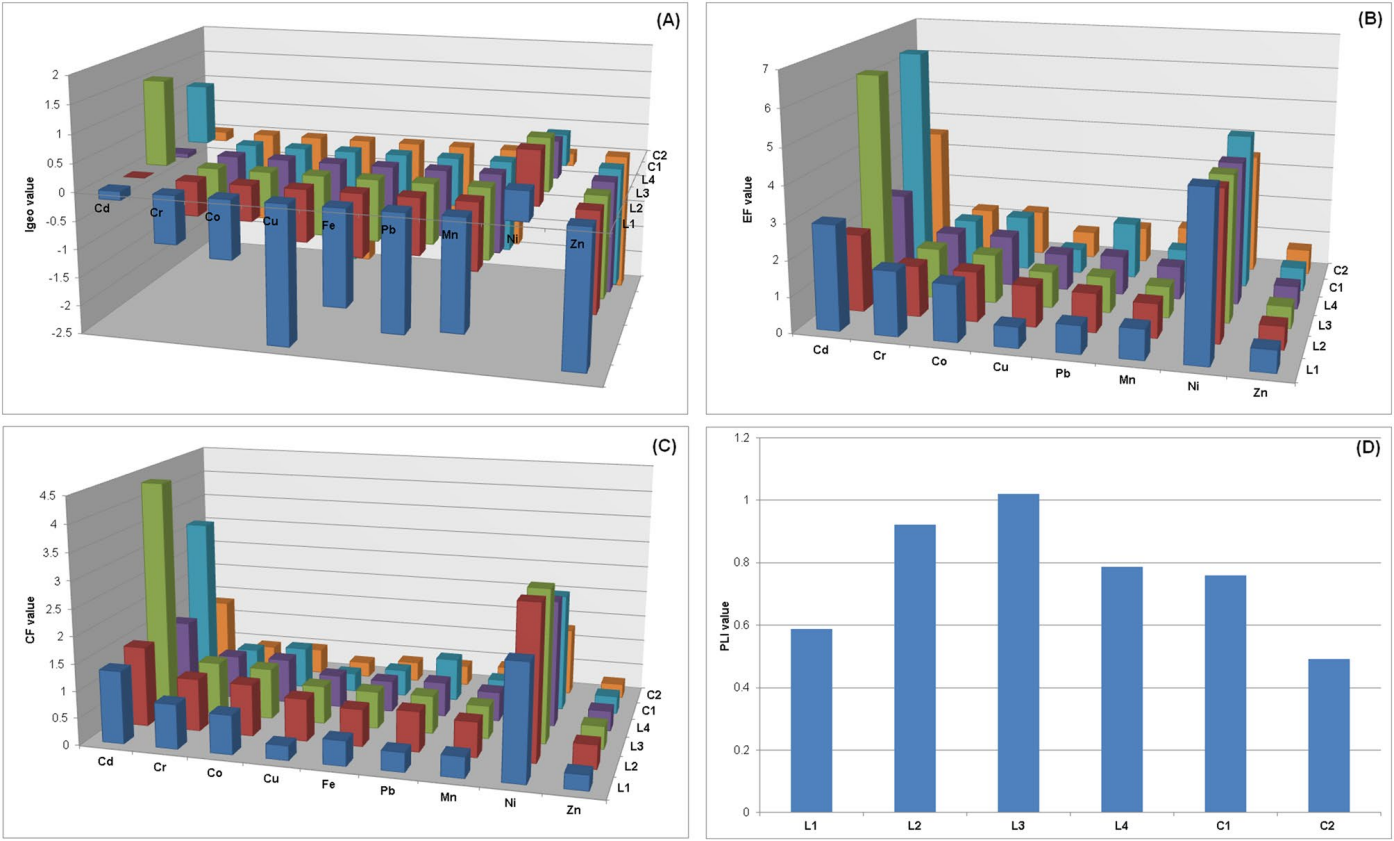
(**A**) Heavy metal geoaccumulation indices (I_geos_), (**B**) enrichment factors (EFs), (**C**) contamination factors (CFs), and (**D**) pollution load indices (PLIs) for Lake Bafa shallow sediment.

The I_geos_ for the sampling points are shown in Fig. 1A. The I_geos_ indicate that sediment at most of the sampling points was uncontaminated (I_geo_ ≤ 0) with heavy metals but that sediment at some sampling points was contaminated with Cd and Ni. Sediment at L1, L2, L3, L4, and C1 was uncontaminated to moderately contaminated with Ni (0 ≤ I_geo_ ≤ 1). Sediment at L3 and C1 was moderately contaminated with Cd (1 ≤ I_geo_ ≤ 2). The mean I_geos_ decreased in the order Ni (0.561) > Cd (0.372) > Co (− 0.955) > Cr (− 0.965) > Pb (− 1.372) > Fe (− 1.477) > Mn (− 1.564) > Cu (− 1.710) > Zn (− 2.106).

The effects of anthropogenic sources of heavy metals on the heavy metal concentrations in the shallow sediments of the lake were assessed by calculating values of EF. Fe was used as a reference element to differentiate between anthropogenic and natural sources, and has previously been used to determine anthropogenic metal enrichment^59–61^. The calculated heavy metal EFs are shown in Fig. 1B. The Cu, Mn, and Zn EFs for all the sampling points were < 1.5, indicating that Cu, Mn, and Zn are not anthropogenic but may have natural sources. The Co, Cr, and Pb EFs were relatively low (1.5 < EF < 3) for some sampling points. The Cd and Ni EFs were relatively high. The Cd EFs for L1, L2, L4, and C2, and the Ni EFs for all the sampling points were moderate (3 < EF < 5), and the Cd EFs for L3 and C1 were very high (5 < EF < 10). The mean EF decreased in the order Ni (4.123) > Cd (3.953) > Co (1.439) > Cr (1.435) > Pb (1.101) > Mn (0.946) > Cu (0.876) > Zn (0.648).

The heavy metal CFs are shown in Fig. 1C. The Co, Cr, Cu, Fe, Mn, Pb, and Zn CFs were all < 1 (indicating a low degree of contamination). The Cd CFs for L1, L2, L4, and C2 and the Ni CFs for all the sampling points indicate moderate contamination (1 < CF < 3), and the Cd CFs for L3 and C1 indicate strong contamination (3 < CF < 6). The mean CFs decreased in the order Ni (2.287) > Cd (2.194) > Co (0.794) > Cr (0.793) > Pb (0.609) > Fe (0.552) > Mn (0.517) > Cu (0.503) > Zn (0.355).

The PLIs were in the range 0.49–1.02 (Fig. 1D). The PLIs for L1, L2, L4, C1, and C2 were < 1, indicating no contamination. The PLI for L3 was > 1, indicating that the sediment at L3 was contaminated with heavy metals, probably from anthropogenic sources.

### Multivariate analytical methods

In order to reduce heavy metal pollution in Lake Bafa, assessment of current pollution alone is not sufficient. It is also important to identify the sources of this pollution in order to establish an effective program of measures. In many previous studies, successful results have been obtained using multivariate analyses, which are known to be effective tools for determining the sources of heavy metal pollution in sediment^62–65^.

Pearson correlation analyses were performed to identify relationships between the concentrations of different heavy metals, and the results are shown in Supplementary Table S1 online. Strong positive correlations were found between the concentrations of some heavy metals, and the strongest relationships, in order of decreasing correlation coefficient, were between the concentrations of Cu and Mn (correlation coefficient 0.999, p < 0.01), Co and Fe (0.997, p < 0.01), Cu and Pb (0.996, p < 0.01), Mn and Zn (0.996, p < 0.01), Cu and Zn (0.993, p < 0.01), Fe and Zn (0.993, p < 0.01), Co and Zn (0.991, p < 0.01), and Pb and Mn (0.990, p < 0.01). However, strong correlations were not found between any of Cd, Cr, and Ni, and other heavy metals.

**Figure 2:**
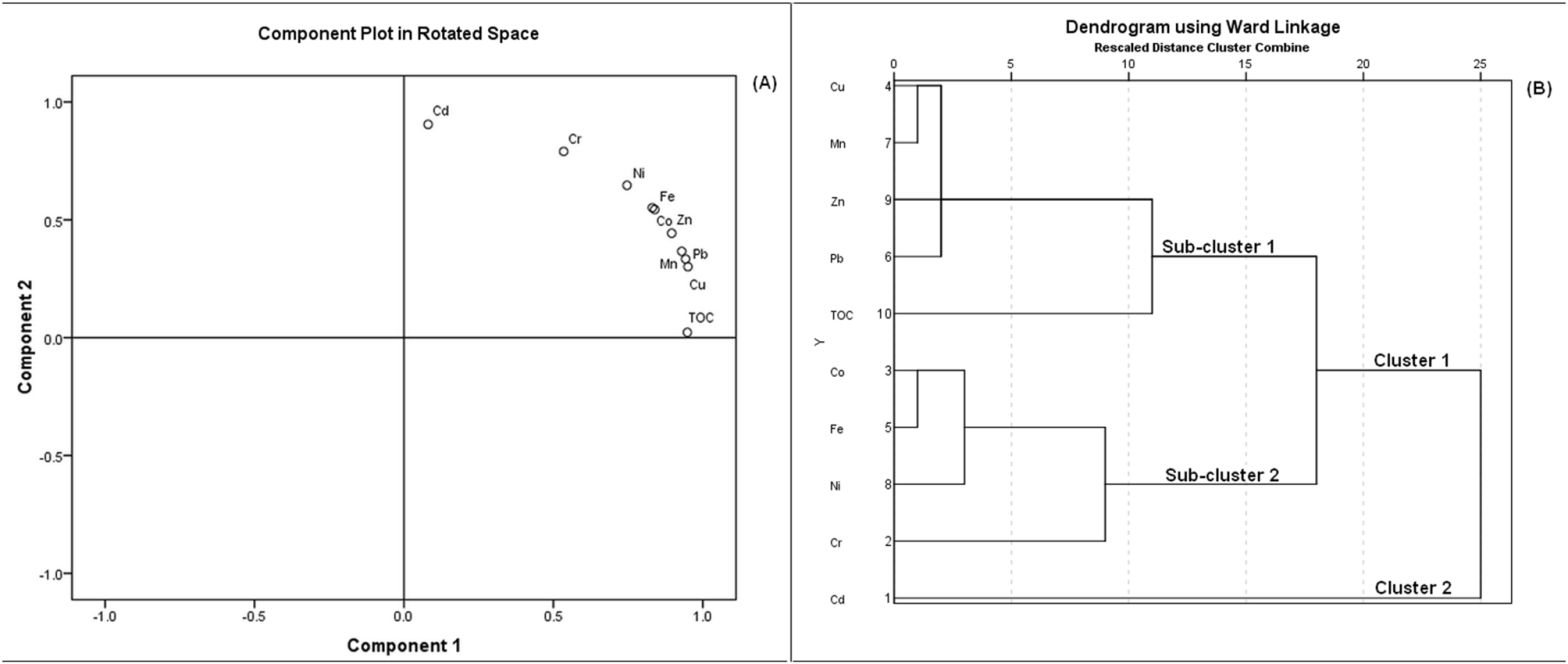
(**A**) Principal component 2 plotted against principal component 1, (**B**) Dendrogram of HCA for heavy metals and TOC in Lake Bafa shallow sediment.

The sources of the heavy metals found in the shallow sediments in Lake Bafa were investigated using PCA and HCA. The TOC content was included in the PCA and HCA to determine whether the heavy metals and organic matter had a common source. The PCA results for the heavy metal concentrations and TOC contents are shown in Supplementary Table S2 online and Fig. 2A. PCA was performed using Varimax rotation with Kaiser Normalization. Results of the PCA indicate that the variables can be grouped into two principal components. Component 1 (PC1) is positively associated with the Co, Cu, Fe, Mn, Ni, Pb, and Zn concentrations and TOC contents. Component 2 (PC2) is associated with the Cd, Cr, and Ni concentrations. PC1 and PC2 explain 66.02% and 29.94%, respectively, of the total variance. Ni has a high loading for both PC1 and PC2.

HCA was performed on standardized data with a Z-scores using Ward’s method and the method of Euclidean distance^66^. The analysed parameters were divided into two major clusters, and the dendrogram showing the HCA results is presented in Fig. 2B. Cluster 1 consists of two sub-clusters. Sub-cluster 1 includes Cu, Mn, Zn, Pb, and TOC, and sub-cluster 2 includes Co, Fe, Ni, and Cr. Cluster 2 consists of only Cd. In general, HCA results were compatible with PCA. HCA gave results that divided the PC1 and PC2 components into clusters with more detailed relationships. Heavy metals in sub-cluster 2 (Cr, Co, Fe, and Ni) contain significant relationships with both components in PCA analysis, especially Ni. PC1 of this group had moderate-to-high positive loadings for Cr (0.534), Co (0.831), Fe (0.839), and Ni (0.746). PC2 of this group was categorized by moderate to high positive loadings for Cr (0.790), Co (0.551), Fe (0.544), and Ni (0.646).

## Discussion

The concentrations and sources of heavy metals in the shallow sediments of Lake Bafa and its tributaries were determined in order to provide data to develop measures to restore the lake. In this study, mean contents of heavy metals in decreasing order were found to be Fe > Mn > Ni > Cr > Zn > Cu > Co > Pb > Cd. These results are in agreement with those of a previous study conducted in the same area^33^. It is also clear that heavy metal concentrations varied spatially, with the highest mean Co, Cu, Mn, Ni, and Zn concentrations on the northern side of the lake (L2), and the highest mean Cd, Cr, and Fe concentrations on the eastern side (L3). The highest mean Pb concentration was found at the entrance channel C1 (north-western side). Although there was no consistent trend in the seasonal variation of heavy metals in the sediment of the lake, it was observed that all concentrations increased in the autumn. The lake was once an embayment of the Aegean Sea, but the development of the Büyük Menderes Delta transformed it into a lake, implying that Lake Bafa can be considered a sediment trap^35^. Currently, sediment transport from the Büyük Menderes River to Lake Bafa is continuous. Given that the heaviest rainfall in the catchment occurs during the autumn, the hydrodynamic conditions of the Büyük Menderes River increase during this time and the sediment transport is more rapid, implying more pollution in terms of the input of heavy metals.

Fe and Mn, which are found naturally in high concentrations in the earth's crust, were detected in the ASVs for the sediments of Lake Bafa. Moreover, Pb and Zn concentrations were measured at levels below the ASVs. In a study carried out by Aydın-Onen et al.^34^, it was revealed that Zn has the ability to accumulate in biota more than in sediment in Lake Bafa. The Pb concentration exceeded the ASV only in the autumn at location C1, which is close to a road with heavy traffic. This is an indicator of sediment transport contaminated by Pb from the highway, as well as from the Büyük Menderes River after heavy rain in the autumn. Seasonal mean Co concentrations in the shallow sediment were found to be higher than the ASVs at almost all sampling points. Co usually reaches aquatic environments by natural means, such as via rock and soil erosion, and is only toxic at very high concentrations^67^. The seasonal mean Cd concentrations for all sampling points were higher than the ASVs. Moreover, Cd concentrations at L3 and C1 were higher than the TEC in autumn and summer, respectively. Relatively high Cd concentrations were detected compared to previous studies^34,35^ in the same area. This variation in concentrations of Cd, which is very toxic in the aquatic ecosystem, is due to the different sampling points and monitoring periods used. Cd has a relatively high solubility in water, and it also tends to bioaccumulate in sediment^68^. Cd pollution in Lake Bafa is caused by protective dyes and fuel used for fishing boats, phosphate fertilizers and pesticides used in agricultural areas, heavy traffic on the Milas-Söke highway, broader fishing activity around the lake, and intense industrial activity in the Büyük Menderes River basin. Concentrations of Cr, Cu and particularly Ni measured in the shallow sediment of Lake Bafa all exceeded SQGs. Seasonal mean Cu concentrations measured in the sediment were found to be above the TEC values only at locations L2 and L3. The Cu concentrations measured within the scope of this study are compatible with those found in previous studies of the sediment in Lake Bafa^33,34^. Cu is a natural mineral that is abundant in nature, quite apart from its widespread use in industry and elsewhere^69^. Its anthropogenic sources are pesticides, fertilizers, corrosion-resistant materials, and domestic wastewater^70^. Apart from this, copper sulphate is the most widely used algicide for preventing diseases in fish breeding^71^. Relatively high Cu concentrations measured in the shallow sediment of the lake stem from domestic wastewater, and from agricultural and aquacultural activities. Ni is present in the natural strata of the Menderes Delta, in which Lake Bafa is located^33,72^. However, the fact that the Ni concentrations are greater than PEC at all sampling points suggests that the origins of the Ni in the Lake Bafa sediment are both natural and anthropogenic. The spread of Ni to the environment from both natural and anthropogenic sources has been noted in air, water and soil^71^, and its anthrophogenic sources are smelting, metal mining, vehicle emissions, fossil fuel burning, household, municipal and industrial waste, fertilizer and organic manure^73^. Ni does not accumulate in biota^74^, but has accumulated in high concentrations in the shallow sediments of Lake Bafa. Seasonal mean Cr concentrations exceeded TEC at all sampling points except C2, and at location L1, Cr was also measured at higher than PEC in autumn. High Cr concentrations were also found in previous studies of the sediment of Lake Bafa^33,34,35^. Its main sources are industrial wastewaters, mainly industrial leather wastewater and domestic wastewater^75^. The main source of Cr pollution in this case is the industrial wastewater originating from the leather industry in Uşak and Aydın Karacasu, reaching the lake via the Büyük Menderes River.

Within the scope of the study, after comparing heavy metal concentrations measured in the shallow sediments of Lake Bafa with threshold values (ASVs, TEC, and PEC), some indices and multivariate statistical analysis were used additionally to support these comparisons. The mean I_geo_ decreased in the order Ni > Cd > Co > Cr > Pb > Fe > Mn > Cu > Zn, the mean EF decreased in the order Ni > Cd > Co > Cr > Pb > Mn > Cu > Zn, and the mean CF decreased in the order Ni > Cd > Co > Cr > Pb > Fe > Mn > Cu > Zn. As can be seen, the heavy metals have the same order in terms of calculated I_geo_, EF, and CF. According to the EFs and CFs, all sampling points were found to be moderately contaminated, whereas according to I_geo_, all sampling points except C2 were found to be uncontaminated to moderately contaminated. In terms of Cd, EF and CF for L3 and C1 were found to be very highly contaminated, while other sampling points were only moderately contaminated. According to I_geo_, L3 and C1 were determined to be uncontaminated to moderately contaminated. When the sediment pollution indices are compared in terms of Ni and Cd, it is seen that the same results were obtained for EF and CF, while relatively low contamination was determined for I_geo_, which indicates that the sediment was uncontaminated with other heavy metals except for Ni and Cd. Regarding CF, all sampling points for Co, Cr, Cu, Fe, Mn, Pb, and Zn were found to have low contamination, while according to EF, Co, Cr, and Pb had low contamination and Cu, Mn, and Zn were found to have come from natural sources. All sediment pollution indices, especially EF and CF, showed generally consistent results.

The PLIs indicate that all the sampling points except L3 (eastern side) were uncontaminated. Sediment at L3 was contaminated with heavy metals from anthropogenic sources. Domestic wastewater from settlements with no sewage infrastructure near L3 could have been the main source of pollutants in this part of the lake. Moreover, the most important sources of heavy metals near L3 are olive oil mills and tourist facilities. Pollution loads from these domestic and industrial wastewaters and solid wastes threaten the water quality in Lake Bafa at least in terms of heavy metals. Pollutants from the Büyük Menderes River enter Lake Bafa near L1, which is also located near sites of intensive agriculture and aquaculture. The PLI values indicate that sediment at L1 was uncontaminated, even though the area around L1 is affected by potential pollutant sources. L1 was in shallow water covered by aquatic plants, which tend to sorb and accumulate heavy metals. For this reason, the worst heavy metal pollution was generally found at the other sampling points.

The results of the PCA indicate that Co, Cu, Fe, Mn, Pb, and Zn predominantly came from similar sources. Cd and Cr came mainly from another common source, and Ni originated from both these sources. Different strong positive correlations were found between the concentrations of Cu, Co, Mn, Fe, Pb, and Zn, and Pearson correlation results support the contention that these heavy metals have common sources. Strong Pearson correlations were not found between any of Cd, Cr, and Ni, and other heavy metals. This indicates that Cd, Cr, and Ni come from sources different from those of other heavy metals^76^. In order to corroborate the results obtained from PCA, HCA was undertaken for heavy metals and TOC. The HCA results were compatible with the results of PCA. Furthermore, using HCA it was possible to divide the 2 components (PC1 and PC2) produced by PCA into other clusters that were both more detailed and more distinct. The HCA results showed that Cd (cluster-1) had no significant correlation with other heavy metals and TOC, which lends support to the view that Cd reached the lake sediment from a different source altogether. It is clear that Cd reaches the lake sediment from anthropogenic sources, taking into account the SQGs and sediment pollution indices as well. Cluster-2 is divided into two clear sub-clusters. Of these, sub-cluster 1 contains heavy metals (Cu, Mn, Zn, and Pb) detected in the lake sediment even using ASVs, as well as TOC. According to the PCA results, Cu, Mn, Zn, Pb, and TOC were determined to have strong relationships with only PC1. These results suggest that Cu, Mn, Zn, and Pb in the shallow sediment are associated with organic matter and transported into the lake whilst attached to organic matter that comes mainly from natural sources. Unlike sub-cluster 1, sub-cluster 2 contains heavy metals, especially Ni, in concentrations that often exceed threshold values and reveal poor sediment quality. In addition, sub-cluster 2 includes Fe, which is abundant in the earth’s crust, and Co, which reaches the aquatic systems in more natural ways^67^. Similar to the Pearson correlation analysis and PCA result, the strongest relationship between Co and Fe was obtained through HCA. Accordingly, it can be said that sub-cluster 2 contains heavy metals that reach the lake shallow sediment from both natural and anthropogenic sources. Heavy metals in sub-cluster 2 (Cr, Co, Fe, and Ni) also show significant relationships with both components in the PCA analysis, especially Ni. As a result of the study carried out by Ergin et al.^77^ for the same study area, it has been determined that the geological layers of the basin in which Bafa Lake is located are naturally rich in Ni and Cr. For heavy metals in sub-cluster 2, Fe and Co reach the lake sediment from another natural source that is not related to organic matter. Ni and Cr, on the other hand, reach the lake sediment mostly from anthropogenic sources, as well as from natural sources associated with Fe and Co. These findings reinforce other results and are also supported by the SQGs and sediment pollution indices described in this study.

Taking all these results into consideration, we conclude that Cd, Cr, Cu, and particularly Ni pose risks to the ecosystem of Lake Bafa. The high Cd, Cr, Cu, and Ni concentrations in the sediments of Lake Bafa are mainly caused by the use of pesticide and fertilizer in agricultural areas, fuel use and the application of corrosion-resistant paint to fishing boats, releases of untreated wastewater from aquaculture facilities, olive oil mills and tourist facilities, and runoff from settlements without sewage systems. Lake Bafa is a potential Ramsar Area because it is home to many types of waterfowl^78^. These highly toxic heavy metals have adverse effects on aquatic organisms and therefore on valuable wildlife in the region. Restoration of Lake Bafa requires the local government to implement measures to prevent heavy metal pollution as a matter of urgency, particularly in relation to Cd, Cr, Cu, and Ni. These restoration studies should start with training carried out with the aim of providing awareness of the harms of toxic chemicals to fishermen, aquaculture workers, and farmers in the region. In order to reveal the full ecological risk caused by these heavy metals, extensive ecotoxicological studies are needed on the responses of the biota of Lake Bafa to these toxic metals.

## Methods

### Study area

The study area was Lake Bafa, which borders the provinces of Aydın and Muğla in southwestern Turkey. Lake Bafa is in the downstream section of the Büyük Menderes River basin, at 37°30ʹ4ʺN and 27°27ʹ37ʺE. The lake was once a bay of the Aegean Sea, but the development of the Büyük Menderes Delta transformed it into a lake. Lake Bafa has a surface area of 72 km^2^, a maximum depth of 25 m, and a shoreline 50 km long. The lake bottom is below sea level, and the water in the lake is brackish. The lake can be considered to be of medium depth. Lake Bafa is a candidate Ramsar Site and has been designated an “important bird area”.

### Sampling sites

**Figure 3:**
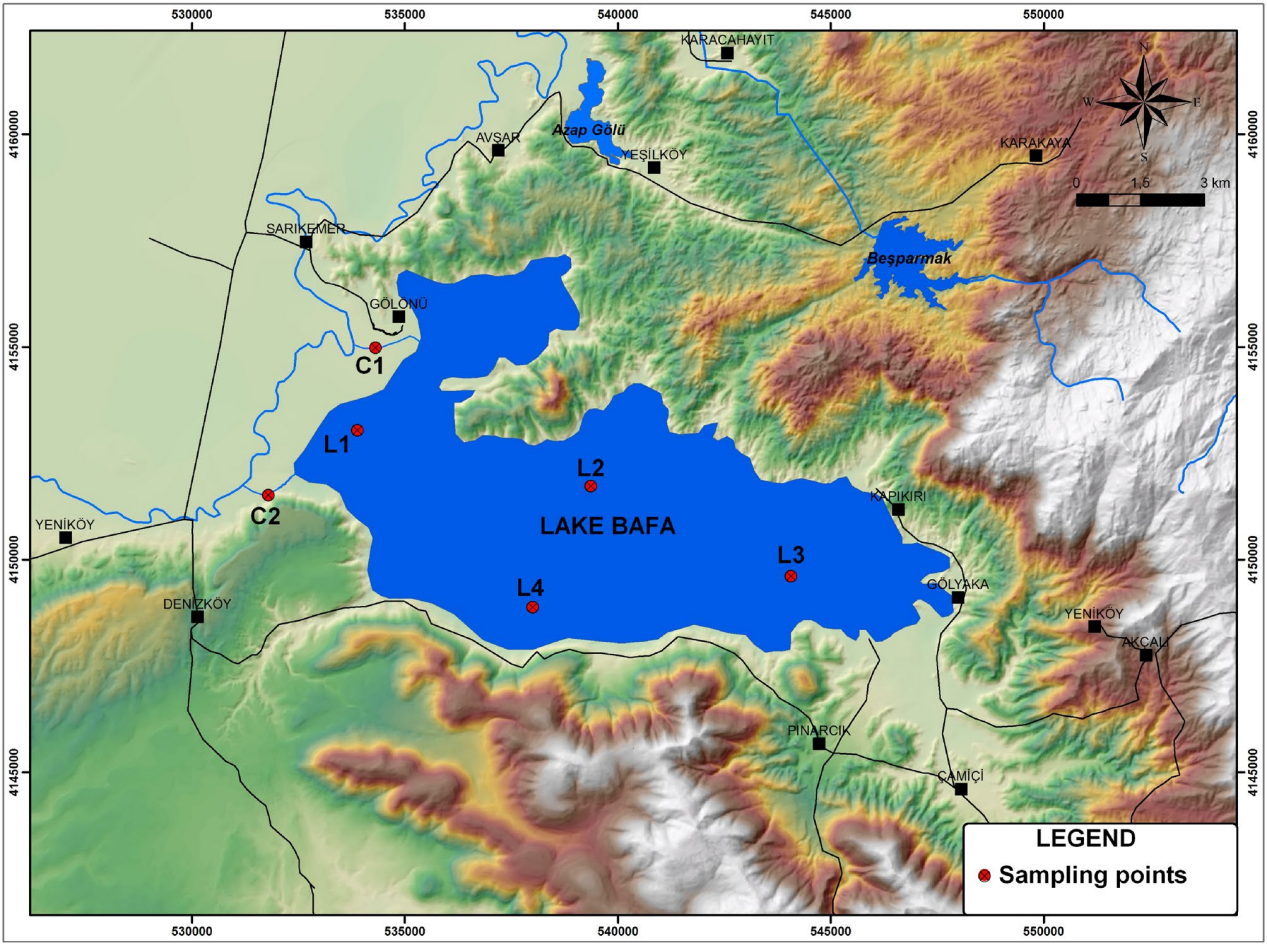
Locations of the sampling points in the study area.

Shallow sediment samples were collected in August and November 2015 and February and May 2016 (i.e., once in each season). Four sediment sampling points (labelled L1–L4) were within the lake, and there was a sediment sampling point in the inflow channel (labelled C1) and another in the outflow channel (labelled C2). The locations of the sediment sampling points are shown in Fig. 3. L1 was in a shallow area (depth 0.7 m) on the western side of the lake. This area was very close to both the Büyük Menderes River inflow channel and the outflow channel. L2 was in the northern part of the lake. The depth at L2 was 23 m. There are no industrial facilities, residential areas, or agricultural areas near the L2 sampling point. L3 was in the eastern part of the lake, and the depth here was 18 m. Domestic wastewater from settlements with no sewage infrastructure near L3 could have been the main source of pollutants in this part of the lake. Solid waste produced by tourist facilities and wastewater from an olive oil processing plant may also supply pollutants to the lake^31^. L4 was in the southern part of the lake; here the depth was 20 m. Heavy metals may be supplied to the part of the lake near L4 by runoff from an intercity highway that crosses the lake. C1 was located to the northwest of the lake in a channel between the Büyük Menderes River and the lake. The area around C1 contains large amounts of agricultural land. C2 was to the southwest of the lake, where there is a sluice. The lake is connected to the Aegean Sea through the outflow channel and the Büyük Menderes River. C2 was very close to industrial fish processing facilities. The area around C2 contains agricultural land.

### Sampling and analytical methods

A shallow sediment (0–2 cm deep) sample was collected from each sampling point at each sampling time using a Van Veen grab sampler. Each sample was immediately packed in an airtight polythene bag and stored at – 20 °C prior to analysis. For heavy metal analysis, sediment samples were dried and then digested with analytical grade reagents following a method described by Kouadia and Trefry^79^. The Cd, Cr, Co, Cu, Fe, Pb, Mn, Ni, and Zn concentrations in the digest were then determined by inductively coupled plasma optical emission spectrometry (ICP-OES 5100, Agilent Inc.). The total organic carbon (TOC) content of each sample was determined using a Shimadzu TOC 5000 automatic analyser (Shimadzu, Kyoto, Japan). Measurement of all samples was carried out in duplicate.

### Data analysis

The I_geos_, EFs, CFs, and PLIs were calculated. The equations used to calculate the pollution indices and the criteria used to evaluate the pollution indices are shown in Table 2. The data were analysed using statistical tests. Pearson correlation analyses were used to identify relationships between the concentrations of different heavy metals, and PCA and HCA were performed to identify the possible sources of heavy metals along the Büyük Menderes basin. All statistical analyses were performed using SPSS software version 16.0 (IBM, Armonk, NY, USA).

**Table 2:** Sediment pollution indices used.

Pollution index	Equation	Evaluation criterias	References
I_geo_	I_geo_ = log_2_(C_n_/1.5 × B_n_)	I_geo_ ≤ 0 (uncontaminated) 0 ≤ I_geo_ ≤ 1 (uncontaminated to moderately contaminated) 1 ≤ I_geo_ ≤ 2 (moderately contaminated) 2 ≤ I_geo_ ≤ 3 (moderately to heavily contaminated) 3 ≤ I_geo_ ≤ 4 (heavily contaminated) 4 ≤ I_geo_ ≤ 5 (heavily to extremely contaminated) I_geo_ ≥ 5 (extremely contaminated)	^37^ ^61^
EF	EF = (M/Fe)_sample_/(M/Fe)_background_	EF > 1.5 (human influence) 1.5 < EF < 3 (minor modification) 3 < EF < 5 (moderate modification) 5 < EF < 10 (severe modification) EF > 10 (very severe modification)	^80^ ^81^ ^61^
CF	CF = C_metal_/C_background_	CF < 1 (low contamination) 1 < CF < 3 (moderate contamination) 3 < CF < 6 (considerable contamination) CF > 6 (high contamination)	^82^ ^14^
PLI	PLI = (CF_1_ × CF_2_ × CF_3_ × … × CF_n_)^1/n^	PLI < 1 (uncontaminated) PLI > 1 (contaminated)	^83^

I_geo_: geoaccumulation index; EF: enrichment factor; CF: contamination factor; PLI: pollution load index; C_n_: measured concentration of element n; B_n_: background concentration of element n; C_metal_: measured concentration of the element; C_background_: background concentration of the element; M/Fe: element to Fe ratio.

## Supplementary information

Supplementary Information.

## Data Availability

Datasets analysed and evaluated during the study are available from the corresponding author on reasonable request.
