# Supplementary material for: Concentrations and sources of heavy metals in shallow sediments in Lake Bafa, Turkey
Source: Sci Rep. 2020 Jul 16;10:11782. doi: 10.1038/s41598-020-68833-2 (PMC7366620; doi:10.1038/s41598-020-68833-2)
Supplement: Supplementary file 1 — Supplementary Information. [file 41598_2020_68833_MOESM1_ESM.pdf]

Concentrations and Sources of Heavy Metals in Shallow Sediments in Lake Bafa, Turkey

Author 1:

Mrs. Fulya Algül

Primary Affiliation: Süleyman Demirel University

Postal Address: Süleyman Demirel University Faculty of Engineering Department of Environmental Engineering Isparta, Turkey

Email: fulyaalgul24@gmail.com

Author 2:

Dr. Mehmet Beyhan* (Corresponding author)

Primary Affiliation: Süleyman Demirel University

Postal Address: Süleyman Demirel University Faculty of Engineering Department of Environmental Engineering Isparta, Turkey

Email: mehmetbeyhan@sdu.edu.tr

Tel: Work +90 246 2111856

Fax +90 246 2111072

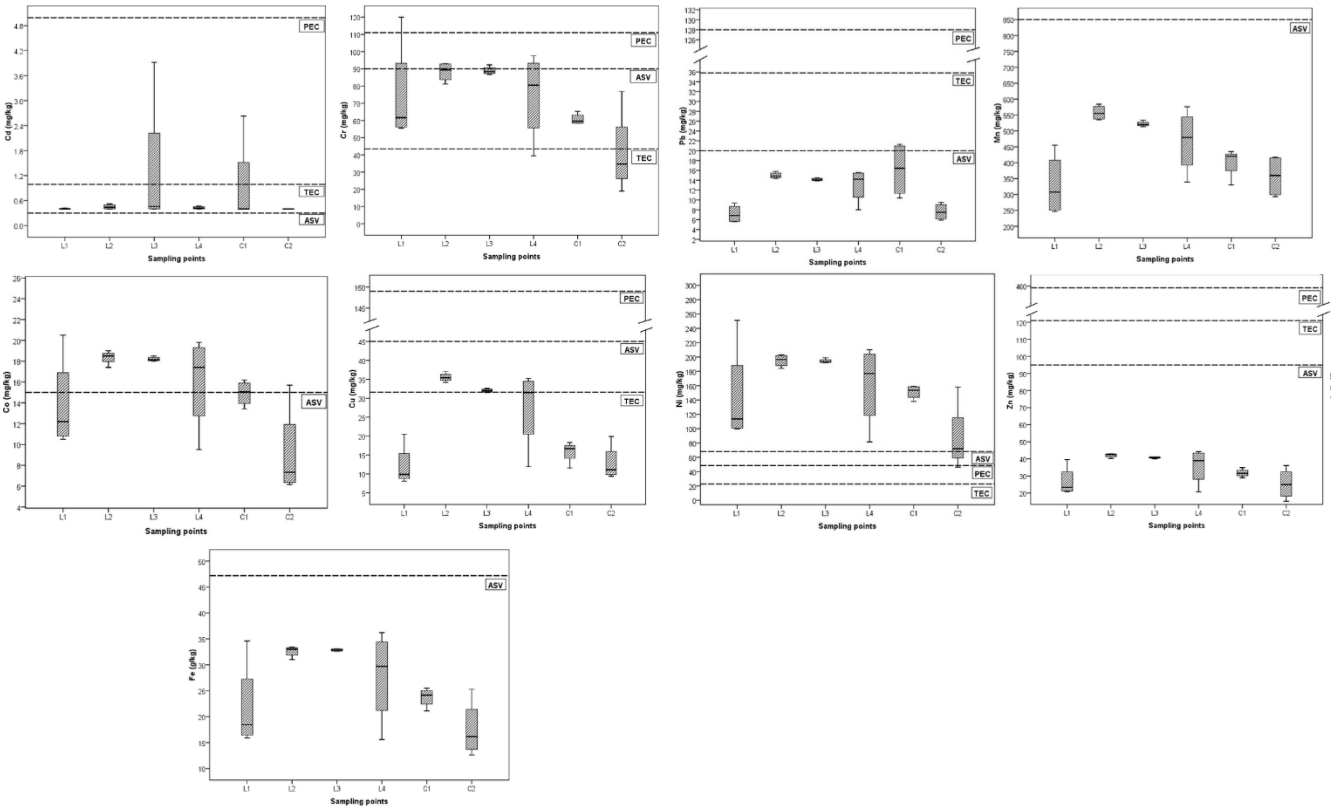

Supplementary Figure S1. Boxplots of the Cd, Cr, Co, Cu, Fe, Pb, Mn, Ni, and Zn concentrations in Lake Bafa shallow sediment, with averaged shale value (ASV) and sediment quality guidelines (threshold effect concentration (TEC) and probable effect concentration (PEC)) shown as dashed horizontal lines on each plot.

Supplementary Table S1. Pearson correlation coefficient matrix for the relationships between the heavy metal concentrations in Lake Bafa shallow sediment. * Significant at the 0.05 level (2-tailed), ** Significant at the 0.01 level (2-tailed).

Variables	Cd	Cr	Co	Cu	Fe	Pb	Mn	Ni	Zn
Cd	1								
Cr	0.633	1							
Co	0.536	0.902	1						
Cu	0.376	0.769	0.968*	1					
Fe	0.560	0.878	0.997**	0.973*	1				
Pb	0.382	0.722	0.950*	0.996**	0.961*	1			
Mn	0.388	0.799	0.978*	0.999**	0.979*	0.990**	1		
Ni	0.579	0.957*	0.988*	0.920	0.978*	0.891	0.937	1	
Zn	0.462	0.838	0.991**	0.993**	0.993**	0.983*	0.996**	0.960*	1

Supplementary Table S2. Rotated principal components for the heavy metal concentrations and total organic carbon content (TOC) (rotation method: varimax with Kaiser normalization).

Variable	PC1	PC2
Cd	0.081	0.905
Cr	0.534	0.790
Co	0.831	0.551
Cu	0.943	0.334
Fe	0.839	0.544
Pb	0.950	0.301
Mn	0.929	0.366
Ni	0.746	0.646
Zn	0.896	0.443
TOC	0.949	0.023
% of variance explained	66.02	29.94
